# A Treatise on Localized Electrization and Its Application to Pathology and Therapeutics

**Published:** 1871-10

**Authors:** 


A Treatise on Localized Electrization and its Applications to Pathology and Therapeutics. By Dr. G. D. Duchenne. Translated from the third edition of the original, by Herbert Tibbets, M. D. With numerous illustrations, and notes, and additions, by the Translator. Philadelphia: Lindsay & Blakiston, Publishers. For sale by Cobb, Andrews & Co., Chicago. Price $3.00.

To all readers of French medical literature, Duchenne's great work on Localized Electrization is already familiar. It is now, however, for the first time rendered accessible to the general profession by means of an English translation.

The original work constitutes not only an exhaustive treatise on the medical uses of electricity, but is also an elaborate exposition of the different diseases in which electricity has proved to be of value as a therapeutical and diagnostic agent. A third edition is now in process of passing through the press, and the volume before us includes all that part of it which had been printed at the time of the investment of Paris by the German army.

The additions by the translator have been made with special reference to the requirements of English medical practitioners, and particularly with a view to facilitate the practice of medical electricity.

